# Faecal microbiome-based machine learning for multi-class disease diagnosis

**DOI:** 10.1038/s41467-022-34405-3

**Published:** 2022-11-10

**Authors:** Qi Su, Qin Liu, Raphaela Iris Lau, Jingwan Zhang, Zhilu Xu, Yun Kit Yeoh, Thomas W. H. Leung, Whitney Tang, Lin Zhang, Jessie Q. Y. Liang, Yuk Kam Yau, Jiaying Zheng, Chengyu Liu, Mengjing Zhang, Chun Pan Cheung, Jessica Y. L. Ching, Hein M. Tun, Jun Yu, Francis K. L. Chan, Siew C. Ng

**Affiliations:** 1 Microbiota I-Center (MagIC), Hong Kong SAR, China; 2 Department of Medicine and Therapeutics, The Chinese University of Hong Kong, Hong Kong SAR, China; 3 Li Ka Shing Institute of Health Sciences, State Key Laboratory of Digestive Disease, Institute of Digestive Disease, The Chinese University of Hong Kong, Hong Kong SAR, China; 4 Center for Gut Microbiota Research, Faculty of Medicine, The Chinese University of Hong Kong, Hong Kong SAR, China; 5 JC School of Public Health and Primary Care, The Chinese University of Hong Kong, Hong Kong SAR, China

**Keywords:** Diagnostic markers, Microbiome

## Abstract

Systemic characterisation of the human faecal microbiome provides the opportunity to develop non-invasive approaches in the diagnosis of a major human disease. However, shared microbial signatures across different diseases make accurate diagnosis challenging in single-disease models. Herein, we present a machine-learning multi-class model using faecal metagenomic dataset of 2,320 individuals with nine well-characterised phenotypes, including colorectal cancer, colorectal adenomas, Crohn’s disease, ulcerative colitis, irritable bowel syndrome, obesity, cardiovascular disease, post-acute COVID-19 syndrome and healthy individuals. Our processed data covers 325 microbial species derived from 14.3 terabytes of sequence. The trained model achieves an area under the receiver operating characteristic curve (AUROC) of 0.90 to 0.99 (Interquartile range, IQR, 0.91–0.94) in predicting different diseases in the independent test set, with a sensitivity of 0.81 to 0.95 (IQR, 0.87–0.93) at a specificity of 0.76 to 0.98 (IQR 0.83–0.95). Metagenomic analysis from public datasets of 1,597 samples across different populations observes comparable predictions with AUROC of 0.69 to 0.91 (IQR 0.79–0.87). Correlation of the top 50 microbial species with disease phenotypes identifies 363 significant associations (FDR < 0.05). This microbiome-based multi-disease model has potential clinical application in disease diagnostics and treatment response monitoring and warrants further exploration.

## Abstract (web summary)

Here, using fecal metagenomics data of 2,320 individuals, the authors develop a microbiome-based machine learning approach showing high accuracy for multi-class disease diagnosis, highlighting its potential application in improving noninvasive diagnostics and monitor responses to therapy.

## Introduction

Recent studies have shown that imbalanced intestinal microbiota, termed “dysbiosis”, contributes to various human diseases^1^. The current development of microbial markers has mostly used binary classifiers^2–5^. Emerging evidence, however, suggests that most health conditions exhibit overlapping gut microbiome signatures^6^, thus single-disease diagnostic models are likely to be confounded by unrelated diseases and may lead to misclassification. Although an attempt has been made to develop a multi-class diagnostic model, heterogeneity, technical bias and batch effects involved in the previous work relying on public datasets for analyses would limit accuracy^7^. Here, we develop the largest single-site dataset to date covering multiple diseases, adopt a machine learning multi-class model to predict different diseases using species-level faecal microbiome profiling, and validate the findings using public metagenome datasets across different populations.

## Results

We performed metagenomic sequencing of faecal samples from 2320 Hong Kong Chinese (mean age 54.9, 48.7% female, Source Data, Supplementary Fig. 1a, see the “Methods” section) consisting of 9 well-characterised disease phenotypes: colorectal cancer (CRC, *n* = 174), colorectal adenomas (CA, *n* = 168), Crohn’s disease (CD, *n* = 200), ulcerative colitis (UC, *n* = 147), irritable bowel syndrome (diarrhoea subtype, IBS-D, *n* = 145), obesity (*n* = 148), cardiovascular disease (CVD, *n* = 143), post-acute COVID-19 syndrome (PACS, *n* = 302) and healthy controls (*n* = 893). In total, we obtained 14.3 terabytes of the sequence at an average depth of 6.15 gigabases for each metagenome and identified 1208 bacterial species. Amongst them, 325 bacterial species had a relative abundance higher than 0.15% and these species were present in over 5% of the subjects (Source Data).

### Shared microbiome signatures across different phenotypes

We observed differences in bacterial diversity (Shannon) and richness (number of species) in different diseases, and we found that both indices vary across phenotypes (Source Data, Supplementary Fig. 1b, c). These results are consistent with a recent meta-analysis^8^, indicating that ecological indices may not be robust indicators of health or disease. Then, we explored associations of microbial composition at the species level with disease phenotypes using a linear model of MaAsLin2 after adjusting for biological and technical confounders (see the “Methods” section). We found a total of 1061 significant associations between these nine phenotypes and 215 bacterial taxa at the species level (FDR < 0.05). Amongst the 215 species, more than 94% were significantly associated with two or more diseases, which is consistent with previous works that numerous signals are shared among different diseases^6,9^ (Source Data, Supplementary Fig. 1d). For instance, *Klebsiella pneumoniae*, a well-characterised opportunistic pathogen^10^, was positively associated with CD, CRC, IBS-D, Obesity, PACS and UC in our cohort, whilst *Roseburia intestinalis*, a promising probiotic with butyrate-producing properties^11^, negatively correlated with these six disease phenotypes (Source Data, Supplementary Fig. 1d). Next, we found that both PCoA analysis based on beta-diversity and random forest (RF) binary classifier could significantly separate all disease phenotypes (Source Data, Supplementary Table 1, Supplementary Fig. 2a–c, all *p* < 0.001). Whilst common microbial signatures were shared across diseases, these findings pointed to the presence of disease-specific microbial composition. However, it is unknown whether binary classifiers can capture these disease-specific signatures. Therefore, we tested the specificity of our trained binary models in unrelated diseases, and the results showed a high misdiagnosis rate (average 0.52, IQR 0.41–0.65, Source Data, Supplementary Fig. 2d). These results suggested that the binary classifier failed to capture real disease-specific features based solely on single disease versus control samples.

### Development of faecal microbiome-based multi-class diagnosis model

**Fig. 1:**
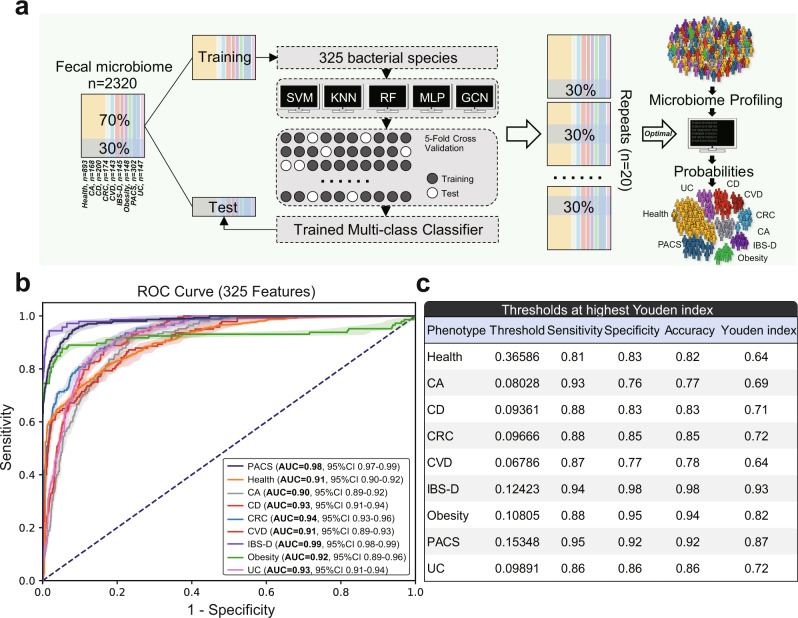
Faecal microbiome-based machine learning for multi-class disease diagnosis. **a** Framework for dataset partition, model training and independent validation. **b** Area under the receiver operating characteristic curve (AUROC, centre for the error bands is median). **c** Performance metric details of the trained random forest multi-class classifier for classifying one phenotype from all others using species-level faecal microbiome data in the independent test set. SVM support vector machine, KNN K-nearest neighbours, RF random forests; MLP multi-layer perceptron, GCN graph convolutional neural network, CA colorectal adenomas, CD Crohn’s disease, CRC colorectal cancer, CVD cardiovascular disease, IBS-D diarrhoea-dominant irritable bowel syndrome, PACS post-acute COVID-19 syndrome, UC ulcerative colitis. Source data are provided as a Source Data file.

Classification tasks in machine learning involving more than two classes are known as “multi-class classification”, which can effectively account for confounding effects of unrelated classes^12^. Based on our cohort of 2320 Hong Kong Chinese, we trained five machine learning multi-class classifiers (RF, K-nearest neighbours (KNN), multi-layer perceptron (MLP), support vector machine (SVM), and graph convolutional neural network (GCN)) to classify different diseases using species-level data from the training set (70% samples with the same class proportions as the cohort) and presented their final performance from the withheld test set (30% samples, Fig. 1a, see the “Methods” section). All these models achieved a mean AUROC of 0.67–0.99 (Interquartile range, IQR 0.81–0.92), suggesting that multi-class disease classification based on the faecal microbiome was feasible (Source Data, Supplementary Fig. 3). Amongst them, the RF multi-class model achieved a mean AUROC of 0.90–0.99 (IQR 0.91–0.94, one versus all others, Fig. 1b) for different disease phenotypes in the test set. The performance of the RF model in the test set significantly outperformed all other models (Source Data, Supplementary Fig. 3b) and was similar to that of the training set (calculated by 5-fold cross-validation, Source Data, Supplementary Fig. 3c), suggesting high integrity of this classifier. Therefore, the RF multi-class model was used for further analyses. At a threshold based on the highest Youden’s Index, the sensitivities of our RF multi-class classifier ranged from 0.81 to 0.95 (IQR 0.87–0.93) at specificities of 0.76 to 0.98 (IQR 0.83–0.95) for different diseases with accuracy from 0.77 to 0.98 (IQR 0.82–0.92, one versus all others, Fig. 1c), highlighting good diagnostic performance. For example, our classifier achieved a mean AUROC of 0.94 for CRC with a sensitivity of 0.88 at a specificity of 0.85 (accuracy 0.85, one versus all others, Fig. 1b, c); this performance was superior to that of our trained binary classifier (CRC versus health, mean AUROC 0.91, Source Data, Supplementary Fig. 2c) and a previously published CRC diagnostic model^2^. Further assessment using predicted probabilities in the test set showed that the trained classifier achieved a mean AUROC of 0.94 for all one versus one classifications (IQR 0.92–0.98, Source Data, Supplementary Fig. 4a) with high sensitivities (IQR 0.88–0.95) and specificities (IQR 0.83–0.94, Source Data, Supplementary Fig. 4b), which supported a superior performance of multi-class model analyses over binary models (Source Data, Supplementary Fig. 2c).

To fully characterise the RF multi-class model, we compared its performance under different split ratios and achieved similar results, suggesting high stability and good predictive power without risk of overfitting (Source Data, Supplementary Fig. 3d). Given that subjects with CRC or colorectal adenomas were older than other subjects (Source Data, Supplementary Fig. 1a), we assessed our model stratified by age and found consistent performance (Source Data, Supplementary Fig. 5). In addition, our model achieved a mean AUROC of 0.87 in distinguishing CRC and colorectal adenomas (Source Data, Supplementary Fig. 4), which supported that effect of age on the model was likely to be negligible. To rule out the possibility that uneven cohort sizes across different diseases may influence the classification performance, we trained a separate RF multi-class classifier by randomly pooling 143 subjects from each disease phenotype (a total of 1287 subjects, 70% training, 30% testing) and found an AUROC of 0.83–0.99 (one versus all others, IQR 0.89–0.96; one versus one, IQR 0.89–0.97; Source Data, Supplementary Fig. 6) which was comparable to the AUROC of 0.90–0.99 in the 2,320 individuals (one versus all others, IQR 0.91–0.94; one versus one, IQR 0.92–0.98; Fig. 1b, Source Data, Supplementary Fig. 4). Importantly, the AUROC values of the model increased with the increasing number of features which suggested again that overfitting based on the 325 selected features was unlikely (Source Data, Supplementary Fig. 7).

### Validation of multi-class model on independent datasets

Then, we integrated 1597 shotgun faecal metagenome data from 12 public datasets from Asia, Europe and North America (Source Data, Supplementary Table 2, Supplementary Fig. 8a). Our RF multi-class classifier showed a mean AUROC of 0.69–0.91 (IQR 0.79–0.87, Source Data, Supplementary Table 3) in classifying different diseases, and generally outperformed all other models (Source Data, Supplementary Fig. 8b). Such performance from an independent validation cohort further confirmed the robustness and generalisability of our model across different populations and geographical locations. To further validate the accuracy of our model, we selected 60 patients who had a complete recovery from COVID-19 infection. Our trained model showed an accuracy of 83.3% (50/60) in classifying these subjects as healthy (Source Data, Supplementary Fig. 8c). These data verified that fully recovered COVID-19 survivors (and without PACS) shared similar gut microbiome profiles as healthy people^13^. Additionally, we also tested our trained RF model on diseases not included in our training dataset, including liver cirrhosis and constipation-dominant IBS datasets (*n* = 60, see the “Methods” section). We found that using our RF multi-class model there were high probabilities whereby prediction cannot be made as they failed the corresponding threshold for most subjects (48/60, Source Data, Supplementary Fig. 8d), and they might be categorised as undetermined. And, the misclassification rate for each phenotype is from 0% (0/60, CA, CVD, IBS-D, Obesity) to 5% (3/60, CD, CRC, PACS, Source Data, Supplementary Fig. 9d), suggesting that our model has a high specificity and accuracy for the nine phenotypes within our cohort with a low risk of misclassification for unrelated diseases.

### Associations between bacterial features and phenotypes

**Fig. 2:**
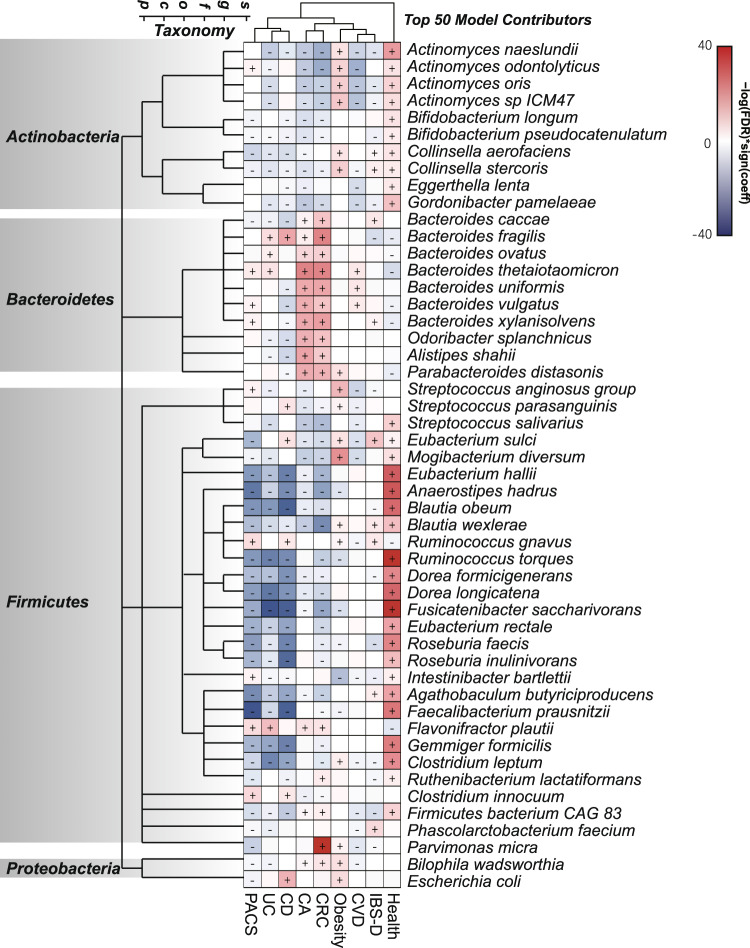
Microbial species associated with health status or different disease phenotypes. The top 50 microbial species contributing to the random forest multi-class classifier were clustered by taxonomy, and different phenotypes were clustered using hierarchical clustering. Associations were coloured by direction of effect (red, positive; blue, negative; *p* < 0.05), with associations significant at FDR < 0.05 marked with a plus (positive correlations) or minus (negative correlations), respectively. The nominal significance (*p*-value) of associations was calculated by MaAsLin 2, and the false discovery rate (FDR) was computed by Benjamini–Hochberg correction. CA colorectal adenomas, CD Crohn’s disease, CRC colorectal cancer, CVD cardiovascular disease, IBS-D diarrhoea-dominant irritable bowel syndrome, PACS post-acute COVID-19 syndrome, UC ulcerative colitis. Source data are provided as a Source Data file.

Next, we correlated the top 50 bacterial species contributing to the model (Source Data, Supplementary Table 4) with different disease phenotypes to identify clues to model interpretability. These top 50 bacterial species achieved a mean AUROC of 0.88 to 0.99 (IQR 0.90–0.93, Source Data, Supplementary Fig. 9a) for different diseases in our test set, and a mean AUROC of 0.67 to 0.90 (IQR 0.78–0.86, Source Data, Supplementary Fig. 9b) in the public dataset. A total of 363 significant associations were found between these 50 species with different disease phenotypes (Hong Kong cohort, FDR < 0.05, Fig. 2). Compared with healthy controls, almost all disease states were associated with a significantly decreased abundance of microbiota from the bacteria phylum of *Firmicutes* or *Actinobacteria* (FDR < 0.05) and a significant increase in *Bacteroidetes* (FDR < 0.05). Imbalance in *Firmicutes*/*Bacteroidetes* ratio had previously been reported primarily in patients with obesity and IBD^14^, but its associations with other diseases have not been reported. Nonetheless, such shared microbial signatures may serve as a basis for distinguishing health and disease. Then, we identified specific microbial signatures that can classify different diseases (Fig. 2). Specifically, the abundance of several bacterial species in *Bacteroidetes* differed significantly between patients with PACS, UC and CD. Subjects with PACS showed a significant increase in abundance of *Bacteroides vulgatus* and *Bacteroides xylanisolvens*, while those with UC were enriched in *Bacteroides ovatus*, and subjects with CD showed significant decreases in *Bacteroides uniformis*, *Bacteroides vulgatus* and *Bacteroides xylanisolvens*, compared with healthy controls. Although patients with CRC and colorectal adenomas shared relatively similar gut bacteria composition, the abundance of *Parvimonas micra* was significantly higher in patients with CRC but not colorectal adenomas, compared to healthy controls, which was consistent with previous findings showing that *Parvimonas micra* can be used as a marker to distinguish CRC from colorectal adenomas^15,16^. For other diseases, microbiome differences were mainly driven by *Actinobacteria*. Subjects with obesity showed increases in *Actinomyces naeslundii*, *Actinomyces odontolyticus* and *Actinomyces oris*, and subjects with IBS-D showed increases in *Collinsella aerofaciens* and *Collinsella stercoris*. We further correlated bacteria and phenotypes in the assembled public dataset, and found that many disease-specific biomarker are stable across datasets, such as *Bacteroides* for UC, *Parvimonas micra* for CRC and *Actinomyces* for obesity (Source Data, Supplementary Fig. 9c). Overall, these results suggest that our model can capture various disease-specific microbial signatures, which may explain the robust diagnostic performance of this multi-class classifier.

## Discussion

Overall, our data showed that the faecal microbiome-based multi-class model for disease diagnosis is feasible. The novelty lies in the high-quality dataset, and superior and reproducible machine-learning methods which are of high clinical relevance. We believe this multi-class model of classifying diseases has potential clinical applications and can serve as a non-invasive way of screening various diseases in clinical practice or for disease risk assessment. Our results also have implications for the potential development of biomarkers for predicting drug response and common treatment strategies using the identified shared or specific marker for multiple diseases. This work has some limitations. Firstly, the disease spectrum of this study is still limited, and the inclusion of more phenotypes can further enhance the value of this multi-class diagnostic tool. Secondly, biological evidence to support the identified microbiome–phenotype associations is limited and future work to delineate the mechanisms of these associations is needed to facilitate our understanding of the role of the shared and disease-specific microbiome in disease pathogenesis. Also, the pooled public dataset did not specify co-morbidities and antibiotic use, thus model performance may vary upon the exclusion of these subjects. Since our model predicts probabilities for multiple diseases simultaneously, it may also apply to multi-disease diagnosis in a single patient. Though we could not validate it at this moment, this hypothesis should be tested in the future.

To our knowledge, we present the largest faecal microbiome datasets with different disease phenotypes and developed a machine learning multi-class model that achieved high performance for disease classification. This non-invasive microbiome-based model could potentially be applied clinically to complement disease diagnostics and treatment response monitoring.

## Methods

### Ethics statement

The study was approved by The Joint Chinese University of Hong Kong – New Territories East Cluster Clinical Research Ethics Committee (The Joint CUHK-NTEC CREC). All subjects provided written informed consent.

### Study population

All participants were recruited and diagnosed at the Prince of Wales Hospital in Hong Kong from January 2017 to March 2022. Subjects with CRC and CA were diagnosed by colonoscopy and confirmed on histology examinations; Subjects with CD and UC were diagnosed based on standard criteria of endoscopy, radiology, and histological examinations. Subjects with IBS were diagnosed according to the ROME III criteria, and endoscopy and enteroscopy were performed to exclude other GI disorders such as IBD, coeliac disease, parasite infestations, or other organic disorders. Obesity was defined as subjects with a body mass index (BMI) of over 28 and with no other medical co-morbidities. Subjects with cardiovascular disease (CVD) were recruited from the public as part of a survey of cardiovascular health in the Hong Kong general population. Subjects underwent carotid ultrasounds to measure intima-media thickness (IMT) of the common, internal, and external carotid arteries (CCA, ICA and ECA, respectively) and carotid bulbs and subjects that had ≥50% stenosis in a single or multiple vessels were regarded as having the risk of CVD. Subjects with post-acute covid-19 syndrome (PACS) were defined as those with at least one persistent symptom or long-term complications of SARS-CoV-2 infection beyond 4 weeks from the viral clearance which could not be explained by an alternative diagnosis, and we assessed the presence of the 30 most commonly reported symptoms post-COVID after illness onset^13,17^ (Source Data, Supplementary Table 5). All subjects with other diseases (apart from the obesity group) had a normal range of BMI of 18.5–22.9. All subjects are on a stable traditional Chinese style diet and are of Han Chinese ethnicity. Patients were excluded if they had the following: age under 18 or over 80; self-reported comorbidities of other diseases; infection with an enteric pathogen; acquired immunodeficiency syndrome; known history of organ dysfunction or failure and abdominal surgery; active malignancy or undergoing radio-chemotherapy; short bowel syndrome; taking drugs commonly known to affect the gut microbiome including proton pump inhibitors, oral anti-diabetics, non-steroidal anti-inflammatory drugs, corticosteroids, laxatives or selective serotonin reactive inhibitors and antibiotics or probiotics use within three months of sample collection; pregnant or breastfeeding; on special diets such as vegetarians.

Healthy controls were recruited during the same recruitment period from the community through advertisement and from the endoscopy centre at the Prince of Wales Hospital and included subjects who had a normal colonoscopy (faecal samples collected before bowel preparation). The exclusion criteria for healthy controls were known complex infections or sepsis; known history of severe organ failure (including decompensated cirrhosis, malignant disease, kidney failure, epilepsy, active serious infection, acquired immunodeficiency syndrome); bowel surgery in the last 6 months (excluding colonoscopy/procedure related to perianal disease); the presence of an ileostomy/stoma; and current pregnancy; any long term drugs for chronic diseases; the use of antibiotics in the last 3 months; the use of laxatives or anti-diarrhoeal drugs in the last 3 months or recent dietary changes (e.g., becoming vegetarian/vegan). Finally, a total of 2320 subjects were recruited. Clinical metadata and dietary data were collected during clinical interviews. Besides, an additional 60 subjects (mean age 53.5, 48.3% female) were prospectively followed up for up to two years after the COVID-19 infection and were confirmed to have fully recovered from the initial infection without any symptoms of PACS. These subjects served as an independent validation cohort and provided serial faecal samples after SARS-CoV-2 clearance.

### Faecal samples

Faecal samples were collected at home by all subjects using tubes prepared by investigators containing preservative media (cat. 63700, Norgen Biotek Corp, Ontario Canada). The Norgen preservative can preserve and allow safe transportation of microbial DNA & RNA at ambient temperature eliminating sample variability. The stool sample was sent to the hospital within 24 h of collection and stored at −80 °C refrigerators until further processing. We have previously shown that data on gut microbiota composition generated from faecal samples collected using this preservative medium was comparable to data obtained from fresh samples that were immediately stored at −80 °C^18^.

### Faecal DNA extraction and sequencing

After removing the preservative media, microbial DNA was isolated with the Qiagen (Hilden, Germany) QIAamp DNA Stool Mini Kit, according to the manufacturer’s instructions. After the quality control procedures by Qubit 2.0, agarose gel electrophoresis, and Agilent 2100, extracted DNA was subject to DNA libraries construction, completed through the processes of end repairing, adding A to tails, purification and PCR amplification, using Nextera DNA Flex Library Preparation kit (Illumina, San Diego, CA). Libraries were subsequently sequenced on our in-house sequencer Illumina NextSeq 550 (150 base pairs paired-end) at the Center for Microbiota Research, The Chinese University of Hong Kong. All samples were in random order for DNA extraction, library construction and sequencing. ZymoBIOMICS Spike-in Control I (High Microbial Load, Cat: D6320-10, ZYMO Research, USA) and ZymoBIOMICS Microbial Community DNA Standard (Cat: D6306-A) were used as positive controls during DNA extraction, library construction, sequencing and quality assessment.

### Microbiome profiling

Raw sequence data were quality filtered using Trimmomatic V.39 to remove the adaptor, low-quality sequences (quality score < 20), and reads shorter than 50 base pairs. Contaminating human reads were filtered using Kneaddata (V.0.10.0, Reference database: GRCh38 p12) with default parameters. Following this, microbiota composition profiles were inferred from quality-filtered forward reads using MetaPhlAn3 version 3.0.14. GNU parallel (v2018) was used for parallel analysis jobs to accelerate data processing. Species whose average abundance and prevalence were <0.15% and 5% were filtered out. Alpha diversity metrics (Shannon diversity, Chao1 richness) were calculated by using the phyloseq package (v1.26.0).

### Microbiome analysis

All statistical analyses were done using R version 4.0.3. The ggpubr package (https://github.com/kassambara/ggpubr) performed nonparametric statistical testing between groups and accounted for multiple hypothesis testing corrections when necessary. Principal coordinates analysis (PCoA) based on beta-diversity (Bray–Curtis distance matrix calculated using relative abundances of microbial species) was used to visualise the clustering of samples based on their species-level compositional profiles. The microbiome composition differences between different phenotypes were calculated by permutational multivariate analysis of variance (PERMANOVA) using distance matrices (adonis) in the adonis function of vegan R package V.2.5–7 with 999 permutations. Associations of specific microbial species with phenotypes were identified using the multivariate analysis by linear models (MaAsLin2) statistical frameworks implemented in the Huttenhower Lab Galaxy instance (http://huttenhower.sph.harvard.edu/galaxy/) with healthy controls as reference. The linear model also included age, sex and technical factors (library DNA concentration, sequencing read depth, sequencing batch) to further correct for potential batch effects and confounders. BMI was not included as apart from the obese group, all subjects from other disease groups had a normal BMI requirement (18.5–22.9) and there was no difference in the BMI across different phenotypes. Benjamini–Hochberg correction was used to control for multiple testing, and results were considered significant at false discovery rate (FDR) < 0.05.

### Random Forest binary classifier

To account for sequencing batch effects for all samples processed in different periods, we performed Combat by SVA package (v3.44.0)^19^ for relative abundance before machine learning model development. Binary sub-cohorts were composed of two phenotypes drawn from the entire cohort. A total of 36 binary sub-cohorts were generated, covering all cross-comparisons of different phenotypes. Machine learning binary classifier used random forest through the Sklearn^20^ library under Python 3.6.7, as this algorithm has been shown to outperform, on average, other learning tools for microbiota data^5^. Normalised abundance table from each binary sub-cohort to train the model. Machine learning models were first trained on the randomly selected training set (70%, 20-times repeated, fivefold-stratified cross-validation) and then were applied to the withheld validation set (30%) to access the final performance. This process was repeated 20 times to obtain a distribution of random forest prediction evaluations on the validation set, and the mean AUROC value was calculated accordingly for the visualisation of and reporting of results.

### Machine learning for diagnosis of multi-diseases

Multi-class models are implemented by Python 3.6.7 using standard libraries that are publicly available: pandas (0.23.4), numpy (1.14.5), scikit-learn (1.1), and matplotlib (2.2.3). For each phenotype, samples were randomly divided into a training set (70% of samples, total *n* = 1724) and a test set for independent evaluation (remaining 30%, total *n* = 696) with balancing class proportions across the cohort, training set and test set. Random forests (RF), *K*-nearest neighbours (KNN), SVM multi-layer perceptron (MLP) and support vector machine (SVM) were used as classifier models for the diagnosis of different phenotypes by using taxonomic profiles at the species level of the faecal microbiome. We implemented the RF multi-class classifier with the following modifications to the default SciKit-learn settings: n_estimaters = 2000 and class_weight = balanced. KNN, SVM and MLP were implemented from SciKit-learn with the default settings. Besides, we reconstructed a graph convolutional neural network (GCN) model based on a published work with the same parameter settings^7^. A nested cross-validation procedure was applied to calculate within-training set accuracy by splitting data into training and test sets for 20-times repeated, fivefold-stratified cross-validation (balancing class proportions across folds). The optimal models selected based on cross-validated results were evaluated in the withheld evaluation dataset as the final performance for predicting different diseases. This process was repeated 20 times to obtain a distribution of random forest prediction evaluations on the validation set, and the mean AUROC and AUPR value was calculated accordingly for the visualisation of results. The highly ranked and frequently selected microbial features were considered predictive signatures for further interpretation. We retrieved prediction performance using the same training datasets.

### Model evaluation

We included AUROC to characterise the model performance as our models initially provided outputs of probabilities for each disease phenotype, and these predicted probabilities were then used to estimate the risk of disease occurrence or absence, which formed a binary status that was analysed to provide an AUROC value. The AUROC is a widely applied metric that considers the trade-offs between sensitivity and specificity at all possible thresholds for comparing the performance across various classifiers with a baseline value of 0.5 for a random classifier. AUPR was provided as a complimentary assessment, which considers the trade-offs between precision (or positive predictive value) and recall (or sensitivity) with a baseline that equals the proportion of positive disease cases in all samples.

### Public data download and processing

For the construction of the external dataset, a total of 1,597 raw shotgun faecal metagenomes were acquired from 12 independently published studies across 11 countries (114 for UC^21–23^, 102 for CD^22–24^, 218 for CVD^25^, 177 for CRC^26–28^, 86 for adenoma^27,28^, 83 for IBS-D^29–31^, 81 for Obesity^32^, and corresponding 736 health controls). Besides, a total of 60 raw shotgun faecal metagenomes from subjects with liver cirrhosis^33^ (*n* = 38, validation cohort from Qin et al.^33^) or constipation-dominant IBS^31^ (IBS-C, *n* = 22) were downloaded to construct an unrelated disease dataset. After downloading, the quality filtration and species-level taxonomic profiling were performed according to the above process.

### Statistics and reproducibility

No statistical method was used to predetermine the sample size. Instead, the study focused on obtaining the largest possible sample size to capture the highest performance of the machine learning multi-class model. 2,320 samples were all successfully sequenced and passed the quality assessment (read depth > 10 million), thus no data were excluded from the analyses. For the machine learning multi-class model, a nested cross-validation procedure was applied to calculate within-training set accuracy by splitting data into training and test sets for 20-times repeated, fivefold-stratified cross-validation (balancing class proportions across folds). The optimal models selected based on cross-validated results were evaluated in the withheld evaluation dataset as the final performance for predicting different diseases. This process was repeated 20 times to obtain a distribution of random forest prediction evaluations on the validation set, and the mean AUROC and AUPR value was calculated accordingly for the visualisation of results. For each phenotype, samples were randomly divided into a training set (70% of samples, total *n* = 1724) and a test set for independent evaluation (remaining 30%, total *n* = 696). Within the training set, a nested cross-validation procedure was applied to calculate within-training set accuracy by randomly splitting data into training and test sets for 20-times repeated, fivefold-stratified cross-validation (balancing class proportions across folds). The study did not include any interventions and thus the conventional blinding (as used in clinical trials or intervention studies) was not relevant to this study.

### Reporting summary

Further information on research design is available in the Nature Research Reporting Summary linked to this article.

## Supplementary information

Supplementary Information

Peer Review File

Reporting Summary

## Data Availability

The raw metagenomes generated in this study have been deposited in the NCBI Sequence Read Archive database under accession code PRJNA841786. The public available raw sequencing data were downloaded through the NCBI Sequence Read Archive using the retrieved accession numbers from cited papers, including DRA006684, DRA008156, ERP008729, ERP005534, ERP023788, ERP021923, PRJEB36140, PRJEB37924, PRJEB33500, PRJNA400072, PRJEB1220, PRJNA429990, PRJEB1220, PRJNA429990, PRJEB15371, and PRJEB6337. The reference database GRCh38.p12 was downloaded from https://www.ncbi.nlm.nih.gov/assembly/GCF_000001405.38. Source data are provided with this paper.

## Source data

Source Data

## Code availability

Codes and scripts developed in this study are all available at the GitHub repository (https://github.com/qsu123/multi_class_diagnosis^34^).
