# Supplementary material for: Faecal microbiome-based machine learning for multi-class disease diagnosis
Source: Nat Commun. 2022 Nov 10;13:6818. doi: 10.1038/s41467-022-34405-3 (PMC9649010; doi:10.1038/s41467-022-34405-3)
Supplement: Supplementary file 1 — Supplementary Information [file 41467_2022_34405_MOESM1_ESM.pdf]

Supplementary Information

Faecal microbiome-based machine learning for multi-class disease diagnosis

Qi Su, Qin Liu, Raphaela Iris Lau, Jingwan Zhang, Zhilu Xu, Yun Kit Yeoh, Thomas WH Leung, Whitney Tang, Lin Zhang, Jessie QY Liang, Yuk Kam Yau, Jiaying Zheng, Chengyu Liu, Mengjing Zhang, Chun Pan Cheung, Jessica YL Ching, Hein M Tun, Jun Yu, Francis KL Chan, Siew C Ng*

*Correspondence:
Siew Chien Ng, Department of Medicine and Therapeutics, Faculty of Medicine, The Chinese University of Hong Kong.
E-mail: siewchienng@cuhk.edu.hk
Tel: (852)3505-1506
Fax: (852) 2647-3852

Supplementary Figures

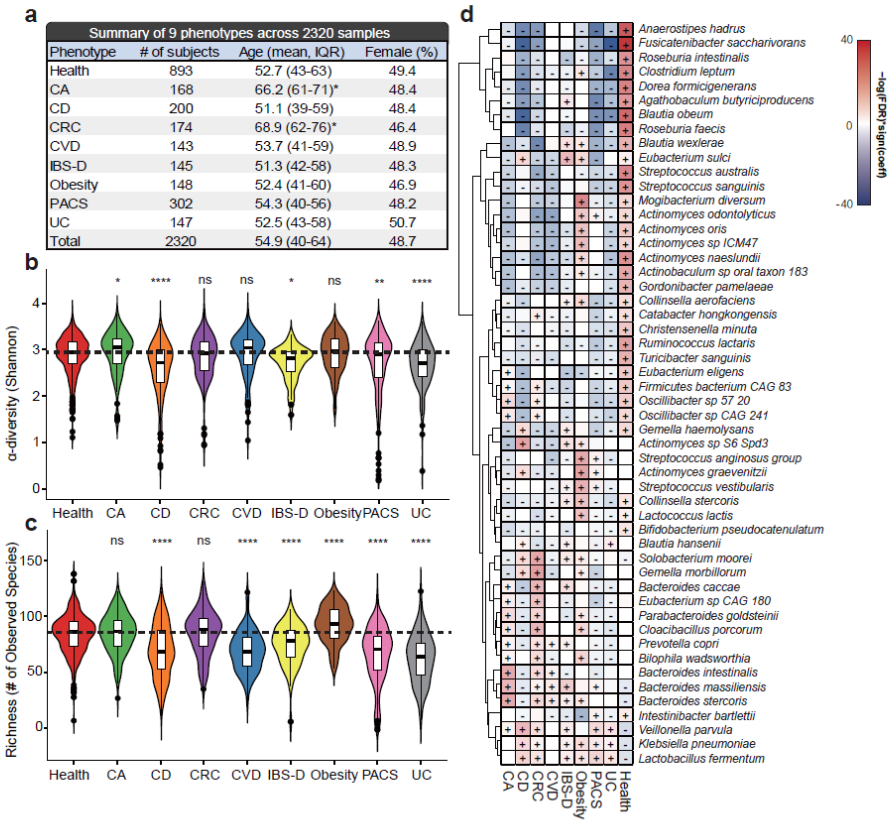

Supplementary Figure 1 Microbiome signatures of health and diseases.
a, Demographics of subjects recruited in this study. Subjects with CA or CRC are older than subjects in other phenotypes ($p<0.05$, Kruskal–Wallis test), but no difference between CA and CRC. No difference was found in gender among different phenotypes. **b,** Alpha diversity (Shannon index) and **c,** Richness (number of observed microbial species) in different phenotypes. P values were calculated by comparing each disease and healthy subjects using MaAsLin2 after adjustment of age, gender and sample processing. Health (n=893), CA (n=168), CD (n=200), CRC (n=174), CVD (n=143), IBS-D (n=145), PACS (n=302), UC (n=147). Centre line is the median, box limits indicate upper and lower quartiles, whiskers show 1.5× interquartile range, points indicate outliers and the outline displays the distribution of the data. **d,** Heat map of microbial species associated with health and mixed diseases. The top 50 microbial species with the highest number of associations were visualised. Associations were coloured by direction of effect (red, positive; blue, negative; $p<0.05$), with associations significant at FDR<0.05 marked with a plus (positive correlations) or minus (negative correlations), respectively. The nominal significance (p value) of associations were calculated by MaAsLin 2, and the FDR was computed by Benjamini-Hochberg correction. CA, colorectal adenomas; CD, Crohn's disease; CRC, colorectal cancer; CVD, Cardiovascular disease; IBS-D, diarrhea-dominant irritable bowel syndrome; PACS, post-acute COVID-19 syndrome; UC, ulcerative colitis.Source data are provided as a Source Data file.

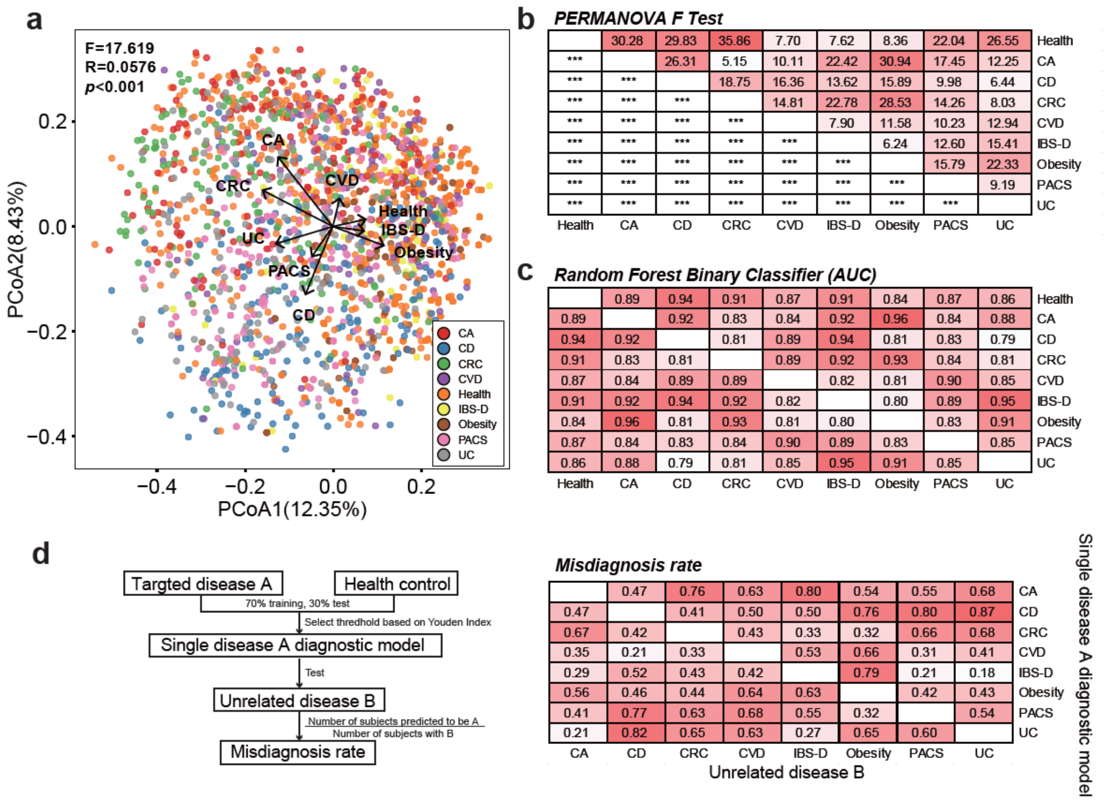

Supplementary Figure 2 Faecal microbiome differences among different phenotypes. a, Bray-Curtis dissimilarity-based PCoA analysis of species-level significantly separated different phenotypes. F, R and P values were calculated by PERMANOVA test with 999 permutations. **b,** Beta-diversity-based F statistics for one phenotype versus one another. F and P values were calculated by PERMANOVA test with 999 permutations. **c,** Area under the receiver operating characteristic curve (AUC) of random forest binary classifiers for one versus one discrimination of multiple phenotypes. **d**, misdiagnosis rate of single disease models in unrelated disease phenotypes. Source data are provided as a Source Data file. CA, colorectal adenomas; CD, Crohn's disease; CRC, colorectal cancer; CVD, Cardiovascular disease; IBS-D, diarrhea-dominant irritable bowel syndrome; PACS, post-acute COVID-19 syndrome; UC, ulcerative colitis.

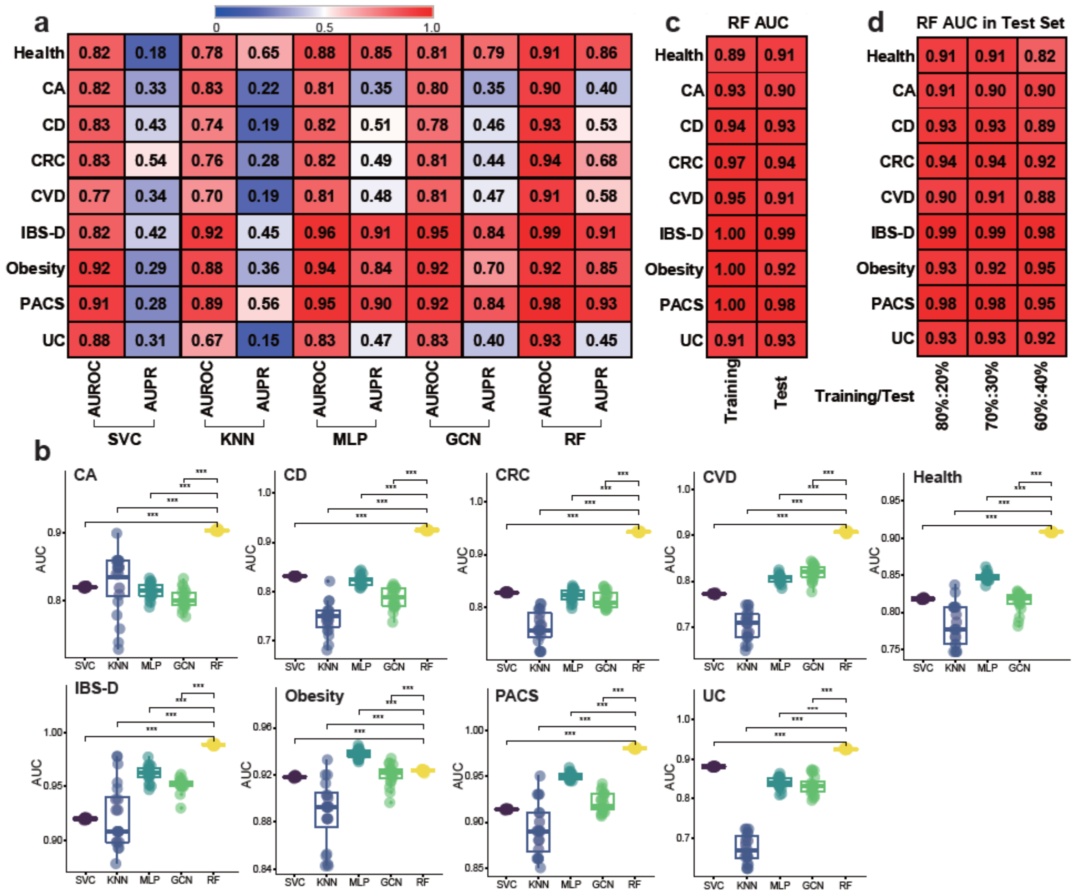

Supplementary Figure 3 Comparison of different classifiers and split ratio for multi-class phenotypes classification using faecal microbiome data at the species level. a, The performance across models was measured using the area under the receiver operating characteristic curve (AUROC) and the area under the precision-recall curve (AUPR) for predicting one phenotype versus all others in the independent test set. **b**, Comparison of AUROC (n=20 repeats) for predicting one phenotype versus all others by different models. P values were calculated by Kruskal–Wallis test. *** represents p<0.001. **c,** AUROC of RF multi-class model in the training set (calculated by 5-fold cross-validation) and test set. Centre line is the median, box limits indicate upper and lower quartiles, whiskers show 1.5× interquartile range, and points indicate outliers. **d,** Performance of the Random Forest multi-class model across different split ratios. SVM, support vector machine; KNN, K-nearest neighbours; RF, Random forests; MLP, multi-layer perceptron; GCN, graph convolutional neural network; CA, colorectal adenomas; CD, Crohn's disease; CRC, colorectal cancer; CVD, Cardiovascular disease; IBS-D, diarrhea-dominant irritable bowel syndrome; PACS, post-acute COVID-19 syndrome; UC, ulcerative colitis. Source data are provided as a Source Data file.

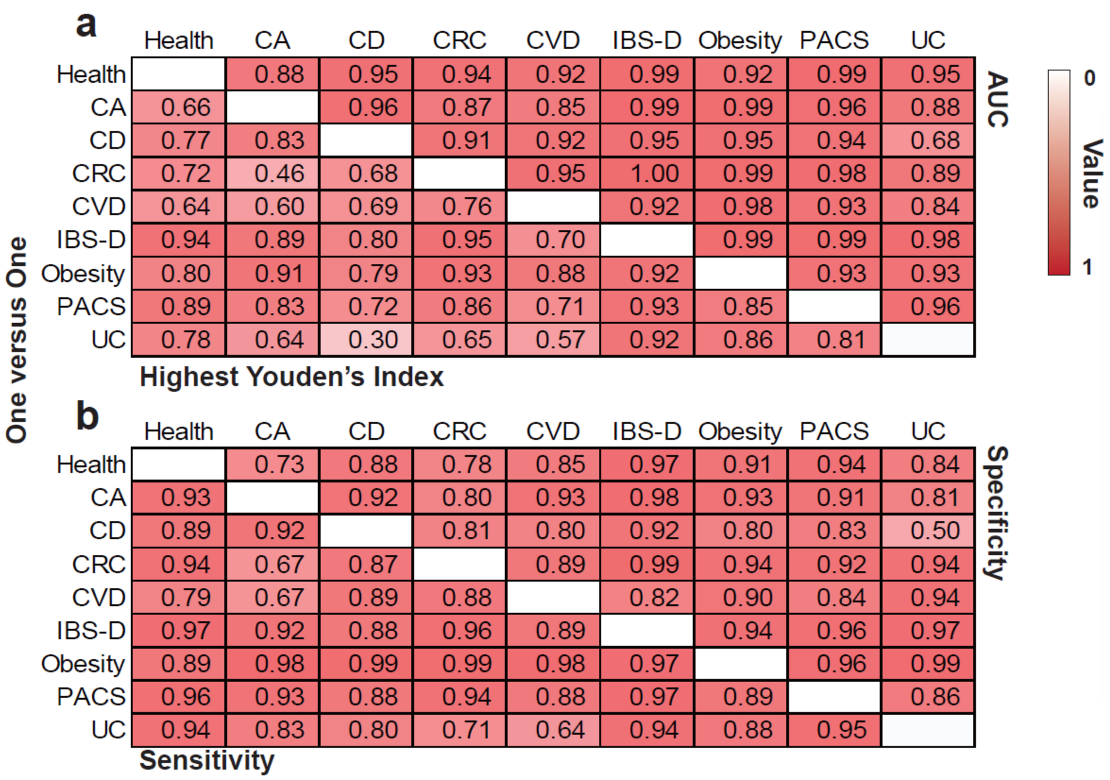

Supplementary Figure 4 Performance of random forest multi-class classifier in one versus one discrimination of multiple phenotypes. a, Area under the receiver operating characteristic curve (AUC) and the highest Youden's index of the random forest multi-class classifier using species-level faecal microbiome data. **b,** sensitivities and specificities selected based on the highest Youden's index for one versus one discrimination of multiple phenotypes. CA, colorectal adenomas; CD, Crohn's disease; CRC, colorectal cancer; CVD, Cardiovascular disease; IBS-D, diarrhea-dominant irritable bowel syndrome; PACS, post-acute COVID-19 syndrome; UC, ulcerative colitis. Source data are provided as a Source Data file.

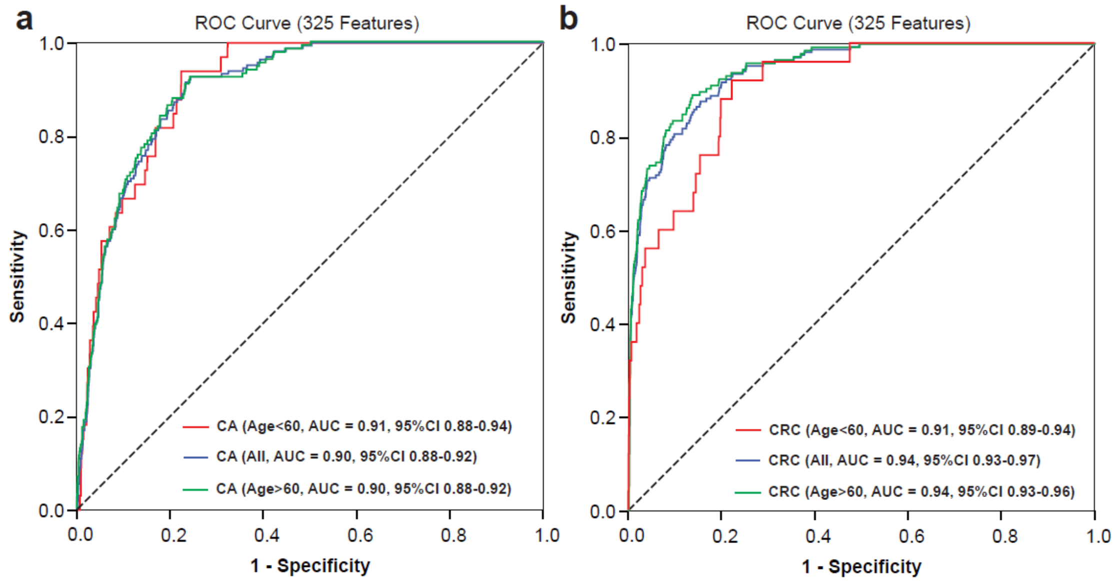

Supplementary Figure 5 Performance of random forest multi-class classifier for colorectal adenomas (a) and colorectal cancer (b) samples stratified by age. CA, colorectal adenomas; CRC, colorectal cancer. Source data are provided as a Source Data file.

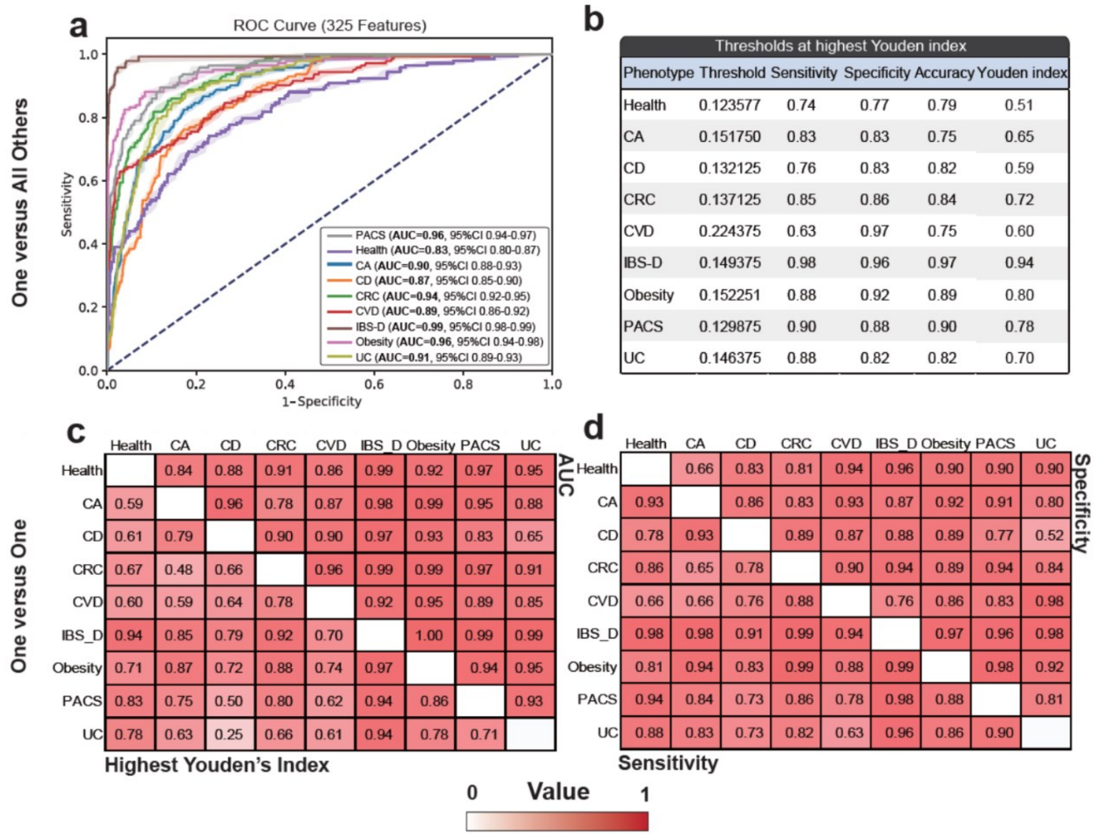

Supplementary Figure 6 Performance of random forest multi-class classifier trained and tested in a balanced sample size cohort. a, Area under the receiver operating characteristic curve (AUROC, centre for the error bands is median) and **b,** Model performance metrics details of random forest multi-class classifier for diagnosing one phenotype versus all others using species-level faecal microbiome data. **c,** AUROC and the highest Youden's index of the random forest multi-class classifier for one versus one discrimination of multiple phenotypes. **d,** Sensitivities and specificities selected based on the highest Youden's index for one versus one discrimination of multiple phenotypes. 143 subjects for each phenotype were randomly selected to form a balanced sample size cohort (a total of 1,287, 70% for training and 30% for test). CA, colorectal adenomas; CD, Crohn's disease; CRC, colorectal cancer; CVD, Cardiovascular disease; IBS-D, diarrhea-dominant irritable bowel syndrome; PACS, post-acute COVID-19 syndrome; UC, ulcerative colitis. Source data are provided as a Source Data file.

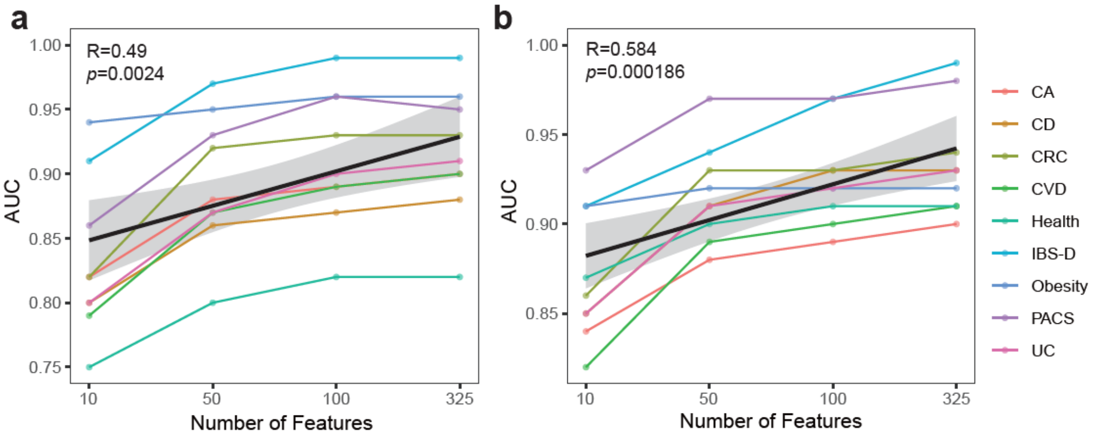

Supplementary Figure 7 Performance of random forest multi-class classifier using a different number of features. The area under the receiver operating characteristic curve (AUROC) of random forest multi-class classifier for diagnosing one phenotype versus all others using the top 10, 50, 100 and all 325 features in (a) the balanced sample size cohort (n=1,287) and (b) the complete cohort (n=2,320). Error bands reflect the 95%CI. R and P values were calculated by Spearman Correlation. CA, colorectal adenomas; CD, Crohn's disease; CRC, colorectal cancer; CVD, Cardiovascular disease; IBS-D, diarrhea-dominant irritable bowel syndrome; PACS, post-acute COVID-19 syndrome; UC, ulcerative colitis. Source data are provided as a Source Data file.

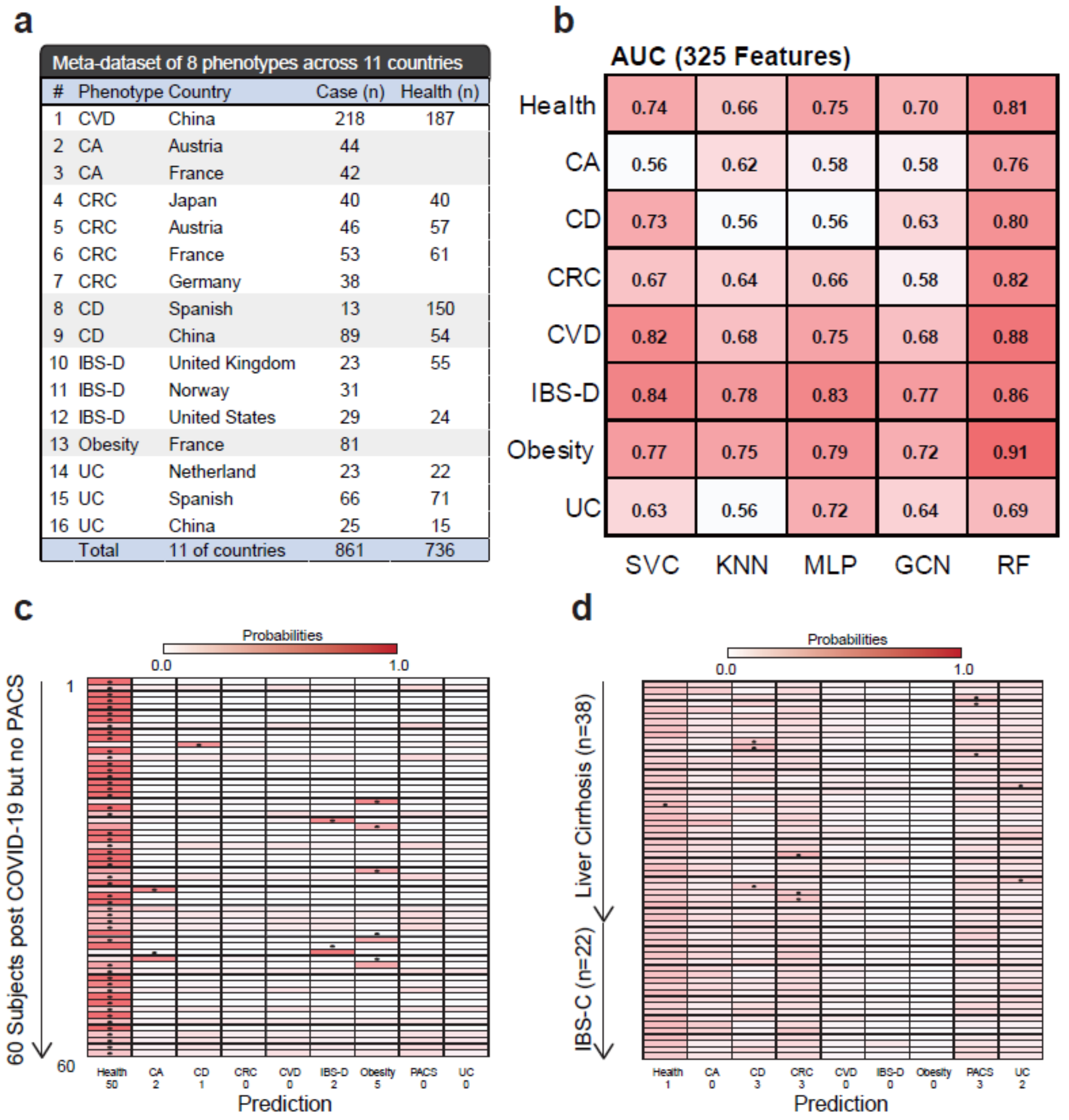

a

#	Phenotype	Country	Case (n)	Health (n)
1	CVD	China	218	187
2	CA	Austria	44	
3	CA	France	42	
4	CRC	Japan	40	40
5	CRC	Austria	46	57
6	CRC	France	53	61
7	CRC	Germany	38	
8	CD	Spanish	13	150
9	CD	China	89	54
10	IBS-D	United Kingdom	23	55
11	IBS-D	Norway	31	
12	IBS-D	United States	29	24
13	Obesity	France	81	
14	UC	Netherland	23	22
15	UC	Spanish	66	71
16	UC	China	25	15
	Total	11 of countries	861	736

Meta-dataset of 8 phenotypes across 11 countries

b

AUC (325 Features)

	SVC	KNN	MLP	GCN	RF
Health	0.74	0.66	0.75	0.70	0.81
CA	0.56	0.62	0.58	0.58	0.76
CD	0.73	0.56	0.56	0.63	0.80
CRC	0.67	0.64	0.66	0.58	0.82
CVD	0.82	0.68	0.75	0.68	0.88
IBS-D	0.84	0.78	0.83	0.77	0.86
Obesity	0.77	0.75	0.79	0.72	0.91
UC	0.63	0.56	0.72	0.64	0.69

Supplementary Figure 8 Independent validations of the performance of random forest multi-class classifier. a, Summary of involved phenotypes and corresponding sample size and source countries in the publicly available cross-regional datasets. **b,** Area under the receiver operating characteristic curve (AUROC) of the trained random forest multi-class classifier for diagnosing one phenotype versus all others in the cross-regional dataset. **c,** Probabilities yielded by the trained random forest multi-class classifier for 60 subjects who completely recovered from COVID-19 without any post-acute COVID-19 syndrome. D, Probabilities yielded by the trained random forest multi-class classifier for 60 subjects with liver cirrhosis (n=38) or constipation-dominant IBS (n=22). The highest probability for each subject that exceed corresponding thresholds are marked with an asterisk. CA, colorectal adenomas; CD, Crohn's disease; CRC, colorectal cancer; CVD, Cardiovascular disease; IBS-D, diarrhea-dominant irritable bowel syndrome; PACS, post-acute COVID-19 syndrome; UC, ulcerative colitis. Source data are provided as a Source Data file.

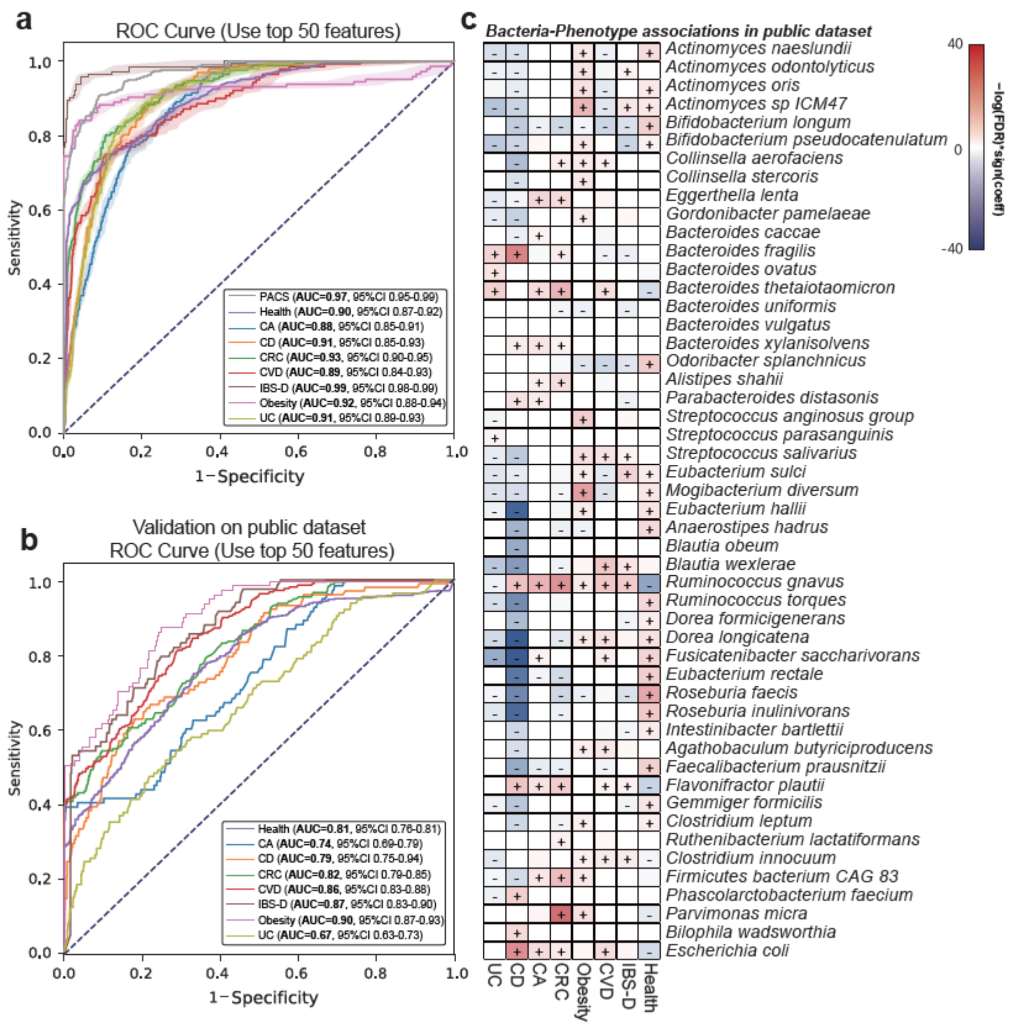

Supplementary Figure 9 Performance of random forest multi-class classifier using top 50 features. a, Area under the receiver operating characteristic curve (AUROC, centre for the error bands is median) of random forest multi-class classifier (using top 50 features) for diagnosing one phenotype versus all others in the independent test set. **b,** Validation of the performance of random forest multi-class classifier (using top 50 features) in the publicly available datasets. **c**, Microbial species associated with health status or different disease phenotypes in the assembled public dataset. The top 50 microbial species contributing to the random forest multi-class classifier were visualized. Associations were coloured by direction of effect (red, positive; blue, negative; $p<0.05$), with associations significant at FDR<0.05 marked with a plus (positive correlations) or minus (negative correlations), respectively. The nominal significance (p value) of associations were calculated by MaAsLin 2, and the FDR was computed by Benjamini-Hochberg correction. CA, colorectal adenomas; CD, Crohn's disease; CRC, colorectal cancer; CVD, Cardiovascular disease; IBS-D, diarrhea-dominant irritable bowel syndrome; UC, ulcerative colitis. CA, colorectal adenomas; CD, Crohn's disease; CRC, colorectal cancer; CVD, Cardiovascular disease; IBS-D, diarrhea-dominant irritable bowel syndrome; PACS, post-acute COVID-19 syndrome; UC, ulcerative colitis. Source data are provided as a Source Data file.

Supplementary Tables

Supplementary Table 1 Beta-diversity-based F statistics (PERMANOVA test) for one phenotype versus another. Part of older subjects with CA and CRC was excluded to eliminate the age difference. F and P values were calculated by PERMANOVA test with 999 permutations.

	8.29	29.83	10.26	7.70	7.62	8.36	22.04	26.55	Health
***		9.84	5.15	4.71	9.37	12.79	6.27	5.43	CA
***	***		5.42	16.36	13.62	15.89	9.98	6.44	CD
***	***	***		5.45	7.92	8.85	5.52	2.36	CRC
***	***	***	***		7.90	11.58	10.23	12.94	CVD
***	***	***	***	***		6.24	12.60	15.41	IBS-D
***	***	***	***	***	***		15.79	22.33	Obesity
***	***	***	***	***	***	***		9.19	PACS
***	***	***	***	***	***	***	***		UC
Health	CA	CD	CRC	CVD	IBS-D	Obesity	PACS	UC	

Notes: *** means p<0.001. CA, colorectal adenomas; CD, Crohn's disease; CRC, colorectal cancer; CVD, Cardiovascular disease; IBS-D, diarrhea-dominant irritable bowel syndrome; UC, ulcerative colitis. CA, colorectal adenomas; CD, Crohn's disease; CRC, colorectal cancer; CVD, Cardiovascular disease; IBS-D, diarrhea-dominant irritable bowel syndrome; PACS, post-acute COVID-19 syndrome; UC, ulcerative colitis.

Supplementary Table 2 Summary of the publicly available metagenome datasets

#	Phenotypes	Country	Year	Author	Case (n)	Control (n)	Data Accession Number
1	UC	Netherland	2018	Franzosa EA et al.	23	22	PRJNA400072
2	UC	Danish/Spanish	2014	Nielsen HB et al.	66	221	PRJEB1220
3	UC	China	2019	Weng YJ et al.	25	15	PRJNA429990
4	CD	Danish/Spanish	2014	Nielsen HB et al.	13		PRJEB1220
5	CD	China	2019	Weng YJ et al.	41		PRJNA429990
6	CD	China	2017	He Q et al.	48	54	PRJEB15371
7	CVD	China	2017	Jie et al.	218	187	ERP023788
8	CRC	Japan	2019	Yachlda et al.	40	40	DRA006684, DRA008156
9	CRC	Austria	2015	Feng et al.	46	57	ERP008729
10	CRC	France/Germany	2014	Zeller et al.	91	61	ERP005534
11	IBS-D	United Kingdom	2021	Vervier K et al.	23	55	ERP021923
12	IBS-D	Norway	2020	Goll et al.	31		PRJEB36140
13	IBS-D	United States	2020	Mars et al.	29	24	PRJEB37924
14	Obesity	France	2020	Meslier et al.	81		PRJEB33500
15	Adenoma	Austria	2015	Feng et al.	44		ERP008729
16	Adenoma	France	2014	Zeller et al.	42		ERP005534

Notes: CA, colorectal adenomas; CD, Crohn's disease; CRC, colorectal cancer; CVD, Cardiovascular disease; IBS-D, diarrhea-dominant irritable bowel syndrome; UC, ulcerative colitis. CA, colorectal adenomas; CD, Crohn's disease; CRC, colorectal cancer; CVD,

Cardiovascular disease; IBS-D, diarrhea-dominant irritable bowel syndrome; PACS, post-acute COVID-19 syndrome; UC, ulcerative colitis.

Supplementary Table 3 Performance details of the trained random forest multi-class classifier using publicly available datasets

Phenotype	Area	Std. Error	Asymptotic Sig.	Asymptotic 95% Confidence Interval	
				Lower Bound	Upper Bound
CA	0.758	0.025	7.690E-16	0.709	0.807
CD	0.798	0.022	5.845E-24	0.755	0.842
CRC	0.819	0.016	1.286E-43	0.787	0.851
CVD	0.879	0.012	1.958E-72	0.856	0.902
Health	0.811	0.011	5.578E-102	0.790	0.832
IBS_D	0.858	0.019	3.653E-28	0.821	0.895
Obesity	0.913	0.013	7.359E-37	0.893	0.942
UC	0.693	0.026	6.806E-12	0.641	0.744

Notes: CA, colorectal adenomas; CD, Crohn's disease; CRC, colorectal cancer; CVD, Cardiovascular disease; IBS-D, diarrhea-dominant irritable bowel syndrome; UC, ulcerative colitis. CA, colorectal adenomas; CD, Crohn's disease; CRC, colorectal cancer; CVD, Cardiovascular disease; IBS-D, diarrhea-dominant irritable bowel syndrome; PACS, post-acute COVID-19 syndrome; UC, ulcerative colitis.

Supplementary Table 4 Importance of scores of the top 50 microbial species in the trained random forest multi-class classifier

**Feature**	**Importance**
Blautia_wexlerae	0.01307182
Fusicatenibacter_saccharivorans	0.012884895
Bacteroides_vulgatus	0.011013169
Agathobaculum_butyriciproducens	0.01085556
Dorea_longicatena	0.010582717
Eubacterium_hallii	0.01009724
Faecalibacterium_prausnitzii	0.009771901
Bacteroides_thetaiotaomicron	0.009741041
Bacteroides_uniformis	0.009703679
Escherichia_coli	0.009688801
Collinsella_aerofaciens	0.009548232
Streptococcus_salivarius	0.009435219
Parvimonas_micra	0.009415211
Eubacterium_sulci	0.009327508
Blautia_obeum	0.009163572

Actinomyces_odontolyticus	0.008993389
Parabacteroides_distasonis	0.008778252
Anaerostipes_hadrus	0.008766194
Flavonifractor_plautii	0.008702062
Bacteroides_fragilis	0.008668667
Mogibacterium_diversum	0.008467032
Dorea_formicigenerans	0.008148955
Ruthenibacterium_lactatiformans	0.007992084
Bilophila_wadsworthia	0.007902528
Roseburia_inulinivorans	0.007901513
Ruminococcus_gnavus	0.007874336
Actinomyces_naeslundii	0.007748365
Bifidobacterium_longum	0.007733054
Actinomyces_sp_ICM47	0.007620109
Gordonibacter_pamelaeae	0.007555373
Intestinibacter_bartlettii	0.007491595
Eubacterium_rectale	0.007419018
Collinsella_stercoris	0.007119509
Eggerthella_lenta	0.007105053
Roseburia_faecis	0.007067439
Streptococcus_parasanguinis	0.007066339
Ruminococcus_torques	0.007000123
Phascolarctobacterium_faecium	0.006936069
Bacteroides_ovatus	0.006908834
Bacteroides_xylanisolvens	0.006900542
Bifidobacterium_pseudocatenulatum	0.006819506
Clostridium_leptum	0.006804848
Actinomyces_oris	0.006801588
Firmicutes_bacterium_CAG_83	0.006658731
Streptococcus_anginosus_group	0.006546908
Odoribacter_splanchnicus	0.006539549
Gemmiger_formicilis	0.006414319
Alistipes_shahii	0.006407032
Bacteroides_caccae	0.006356583

Supplementary Table 5 Questionnaire used for post-acute COVID-19 symptom assessment

**Symptoms**	**Yes/No**	**Duration**
Fever		
Chills		
Cough		
Sputum Production		
Sore throat		
Congested or runny nose		
Fatigue		
Joint pain		
Muscle pain		
Shortness of breath		
Headache		
Dizziness		
Nausea		
Vomiting		
Diarrhoea		
Loss of taste		
Loss of smell		
Abdominal pain		
Epigastric pain		
Difficulty in concentration		
Inability to exercise		
Difficulty in sleeping		
Anxiety		
Sadness		
Memory problem		
Chest pain		
Palpitations		
Night sweats		
Hair loss		
Blurred vision		
Any other symptoms		
