# Supplementary material for: Faecal microbiome-based machine learning for multi-class disease diagnosis
Source: Nat Commun. 2022 Nov 10;13:6818. doi: 10.1038/s41467-022-34405-3 (PMC9649010; doi:10.1038/s41467-022-34405-3)
Supplement: Supplementary file 3 — Reporting Summary [file 41467_2022_34405_MOESM3_ESM.pdf]

nature portfolio

Corresponding author(s): Siew C Ng

Last updated by author(s): 13-10-2022

Reporting Summary

Nature Portfolio wishes to improve the reproducibility of the work that we publish. This form provides structure for consistency and transparency in reporting. For further information on Nature Portfolio policies, see our Editorial Policies and the Editorial Policy Checklist.

Statistics

For all statistical analyses, confirm that the following items are present in the figure legend, table legend, main text, or Methods section.

n/a	Confirmed	
☐	☒	The exact sample size (*n*) for each experimental group/condition, given as a discrete number and unit of measurement
☐	☒	A statement on whether measurements were taken from distinct samples or whether the same sample was measured repeatedly
☐	☒	The statistical test(s) used AND whether they are one- or two-sided<br>*Only common tests should be described solely by name; describe more complex techniques in the Methods section.*
☐	☒	A description of all covariates tested
☐	☒	A description of any assumptions or corrections, such as tests of normality and adjustment for multiple comparisons
☐	☒	A full description of the statistical parameters including central tendency (e.g. means) or other basic estimates (e.g. regression coefficient) AND variation (e.g. standard deviation) or associated estimates of uncertainty (e.g. confidence intervals)
☐	☒	For null hypothesis testing, the test statistic (e.g. *F*, *t*, *r*) with confidence intervals, effect sizes, degrees of freedom and *P* value noted<br>*Give P values as exact values whenever suitable.*
☒	☐	For Bayesian analysis, information on the choice of priors and Markov chain Monte Carlo settings
☒	☐	For hierarchical and complex designs, identification of the appropriate level for tests and full reporting of outcomes
☐	☒	Estimates of effect sizes (e.g. Cohen's *d*, Pearson's *r*), indicating how they were calculated

Our web collection on statistics for biologists contains articles on many of the points above.

Software and code

Policy information about availability of computer code

Data collection	For full details see Methods; Trimmomatic (v39), Kneaddata (v0.10.0), MetaPhlAn3 (v3.0.14), GNU parallel (v2018) were used to process the microbiome sequencing data.
Data analysis	Open-source codes and scripts used for the microbiome analyses or figures are available at the GitHub repository (https://github.com/qsu123/multi_class_diagnosis). All machine learning data processing and modeling were conducted on Python 3.6.7 using standard libraries that are publicly available: pandas (v0.23.4), numpy (v1.14.5), scikit-learn (v1.1), and matplotlib (v2.2.3). Other software used: Microsoft Excel (v16.0.15225.20172), R version 4.0.3, R packages: Phyloseq (v1.26.0), vegan (v2.5-7), MaAsLin2 (v1.7.3), ggpubr (v0.4.0.999).

For manuscripts utilizing custom algorithms or software that are central to the research but not yet described in published literature, software must be made available to editors and reviewers. We strongly encourage code deposition in a community repository (e.g. GitHub). See the Nature Portfolio guidelines for submitting code & software for further information.

Data

Policy information about availability of data

All manuscripts must include a data availability statement. This statement should provide the following information, where applicable:

- Accession codes, unique identifiers, or web links for publicly available datasets
- A description of any restrictions on data availability
- For clinical datasets or third party data, please ensure that the statement adheres to our policy

The raw metagenomes generated in this study have been deposited in the NCBI Sequence Read Archive database under accession code PRJNA841786 (https://www.ncbi.nlm.nih.gov/sra/?term= PRJNA841786). The public available raw sequencing data were downloaded through the NCBI Sequence Read Archive using the retrieved accession numbers from cited papers, including DRA006684(https://www.ncbi.nlm.nih.gov/sra/?term=DRA006684), DRA008156(https://www.ncbi.nlm.nih.gov/sra/?term=DRA008156), ERP008729(https://www.ncbi.nlm.nih.gov/sra/?term=ERP008729), ERP005534(https://www.ncbi.nlm.nih.gov/sra/?term=ERP005534), ERP023788(https://www.ncbi.nlm.nih.gov/sra/?term=ERP023788), ERP021923(https://www.ncbi.nlm.nih.gov/sra/?term=ERP021923), PRJEB36140(https://www.ncbi.nlm.nih.gov/sra/?term=PRJEB36140), PRJEB37924(https://www.ncbi.nlm.nih.gov/sra/?term=PRJEB37924), PRJEB33500(https://www.ncbi.nlm.nih.gov/sra/?term=PRJEB33500), PRJNA400072(https://www.ncbi.nlm.nih.gov/sra/?term=PRJNA400072), PRJEB1220(https://www.ncbi.nlm.nih.gov/sra/?term=PRJEB1220), PRJNA429990(https://www.ncbi.nlm.nih.gov/sra/?term=PRJNA429990), PRJEB1220(https://www.ncbi.nlm.nih.gov/sra/?term=PRJEB1220), PRJNA429990(https://www.ncbi.nlm.nih.gov/sra/?term=PRJNA429990), PRJEB15371(https://www.ncbi.nlm.nih.gov/sra/?term=PRJEB15371), and PRJEB6337(https://www.ncbi.nlm.nih.gov/sra/?term=PRJEB6337). The reference database GRCh38.p12 was downloaded from https://www.ncbi.nlm.nih.gov/assembly/GCF_000001405.38. Source data are provided with this paper.

Human research participants

Policy information about studies involving human research participants and Sex and Gender in Research.

Reporting on sex and gender

2,320 Hong Kong Chinese individuals were involved in this study (average age 54.9, 48.7% female). The information was collected during clinical interviews and verified from the clinical management system (CMS) of hospital authority in Hong Kong

Population characteristics

2,320 Hong Kong Chinese individuals were involved in this study (average age 54.9, 48.7% female), consisting of nine well-characterized disease phenotypes: colorectal cancer (CRC, n=174), colorectal adenomas (CA, n=168), Crohn's disease (CD, n=200), ulcerative colitis (UC, n=147), irritable bowel syndrome (IBS, diarrhea subtype, n=145), obesity (n=148), atherosclerotic cardiovascular disease (CVD, n=143), post-acute COVID-19 syndrome (PACS, n=302), and healthy controls (n=893).

Recruitment

All participants were recruited at the Prince of Wales Hospital in Hong Kong from January 2017 to March 2022. All participating patients strictly adhere to recruitment and exclusion criteria to avoid self-selection bias or other biases. Subjects with CRC and CA were diagnosed by colonoscopy and confirmed on histology examinations; Subjects with CD and UC were diagnosed based on standard criteria of endoscopy, radiology, and histological examinations. Subjects with IBS were diagnosed according to the ROME ? criteria, and endoscopy and enteroscopy were performed to exclude other GI disorders such as IBD, coeliac disease, parasite infestations, or other organic disorders. Obesity was defined as subjects with a body mass index (BMI) of over 28 and with no other medical co-morbidities. Subjects with cardiovascular disease (CVD) were recruited from the public as part of a survey of cardiovascular health in the Hong Kong general population. Subjects underwent carotid ultrasounds to measure intima-media thickness (IMT) of the common, internal, external carotid arteries (CCA, ICA and ECA, respectively) and carotid bulbs and subjects that had ≥50% stenosis in a single or multiple vessels were regarded as having the risk of CVD. Subjects with post-acute covid-19 syndrome (PACS) were defined as those with at least one persistent symptom or long-term complications of SARS-CoV-2 infection beyond 4weeks from the viral clearance which could not be explained by an alternative diagnosis, and we assessed the presence of the 30 most commonly reported symptoms post-COVID after illness onset. All subjects with other diseases (apart from the obesity group) had a normal range of BMI of 18.5 to 22.9. All subjects are on stable traditional Chinese style diet and are Han Chinese ethnicity. Patients were excluded if they had the following: age under 18 or over 80; self-reported comorbidities of other diseases; infection with an enteric pathogen; acquired immunodeficiency syndrome; known history of organ dysfunction or failure and abdominal surgery; active malignancy or undergoing radio-chemotherapy; short bowel syndrome; taking drugs commonly known to affect the gut microbiome including proton pump inhibitors, oral anti-diabetics, non-steroidal anti-inflammatory drugs, corticosteroids, laxatives or selective serotonin reactive inhibitors and antibiotics or probiotics use within three months of sample collection; pregnant or breastfeeding; on special diets such as vegetarians.

Healthy controls were recruited during the same recruitment period from the community through advertisement and from the endoscopy centre at the Prince of Wales Hospital and included subjects who had a normal colonoscopy (faecal samples collected before bowel preparation). All participating healthy controls strictly adhere to exclusion criteria to avoid self-selection bias or other biases. The exclusion criteria for healthy controls were known complex infections or sepsis; known history of severe organ failure (including decompensated cirrhosis, malignant disease, kidney failure, epilepsy, active serious infection, acquired immunodeficiency syndrome); bowel surgery in the last 6 months (excluding colonoscopy/procedure related to perianal disease); the presence of an ileostomy/stoma; and current pregnancy; any long term drugs for chronic diseases; the use of antibiotics in the last 3 months; the use of laxatives or anti-diarrheal drugs in the last 3 months or recent dietary changes (e.g., becoming vegetarian/vegan). Finally, a total of 2,320 subjects were recruited. Clinical metadata and dietary data were collected during clinical interviews. Besides, an additional 60 subjects (mean age 53.5, 48.3% female) were prospectively followed-up for up to two years after the COVID-19 infection and were confirmed to have fully recovered from the initial infection without any symptoms of PACS. These subjects served as an independent validation cohort and provided serial faecal samples after SARS-CoV-2 clearance.

Ethics oversight	The study was approved by The Joint Chinese University of Hong Kong – New Territories East Cluster Clinical Research Ethics Committee (The Joint CUHK-NTEC CREC). All subjects provided written informed consent.

Note that full information on the approval of the study protocol must also be provided in the manuscript.

Field-specific reporting

Please select the one below that is the best fit for your research. If you are not sure, read the appropriate sections before making your selection.

☒ Life sciences ☐ Behavioural & social sciences ☐ Ecological, evolutionary & environmental sciences

For a reference copy of the document with all sections, see nature.com/documents/nr-reporting-summary-flat.pdf

Life sciences study design

All studies must disclose on these points even when the disclosure is negative.

Sample size	Sample size calculation was not performed before the study. Instead the study focused on obtaining the largest possible sample size to capture the highest performance of the machine learning multi-class model.
Data exclusions	All 2,320 samples were successfully sequenced and passed the quality assessment (read depth > 10 million), thus no were excluded from the analyses.
Replication	For the machine learning multi-class model, a nested cross-validation procedure was applied to calculate within-training set accuracy by splitting data into training and test sets for 20-times repeated, fivefold-stratified cross-validation (balancing class proportions across folds). The optimal models selected based on cross-validated results were evaluated in the withheld evaluation dataset as the final performance for predicting different diseases. This process was repeated 20 times to obtain a distribution of random forest prediction evaluations on the validation set, and the mean AUROC and AUPR value was calculated accordingly for visualization of results.<br><br>For independent validation using publicly available datasets, we integrated 1,597 shotgun stool metagenome data from 12 published studies from 11 countries, covering Asia, Europe and North America, and our trained model showed satisfactory performance.
Randomization	For each phenotype, samples were randomly divided into a training set (70% of samples, total n=1,724) and a test set for independent evaluation (remaining 30%, total n=696). Within the training set, a nested cross-validation procedure was applied to calculate within-training set accuracy by randomly splitting data into training and test sets for 20-times repeated, fivefold-stratified cross-validation (balancing class proportions across folds).
Blinding	The conventional blinding (as used in clinical trials or intervention studies) was not relevant for this study because this study did not include any interventions.

Reporting for specific materials, systems and methods

We require information from authors about some types of materials, experimental systems and methods used in many studies. Here, indicate whether each material, system or method listed is relevant to your study. If you are not sure if a list item applies to your research, read the appropriate section before selecting a response.

Materials & experimental systems

n/a	Involved in the study	
☒	☐	Antibodies
☒	☐	Eukaryotic cell lines
☒	☐	Palaeontology and archaeology
☒	☐	Animals and other organisms
☒	☐	Clinical data
☒	☐	Dual use research of concern

Methods

n/a	Involved in the study	
☒	☐	ChIP-seq
☒	☐	Flow cytometry
☒	☐	MRI-based neuroimaging
